# Development of terphenyl-2-methyloxazol-5(4*H*)-one derivatives as selective reversible MAGL inhibitors

**DOI:** 10.1080/14756366.2017.1375484

**Published:** 2017-09-22

**Authors:** Carlotta Granchi, Isabella Caligiuri, Eleonora Bertelli, Giulio Poli, Flavio Rizzolio, Marco Macchia, Adriano Martinelli, Filippo Minutolo, Tiziano Tuccinardi

**Affiliations:** a Department of Pharmacy, University of Pisa, Pisa, Italy;; b Unit of Pathology, Department of Molecular Biology and Translational Research, National Cancer Institute and Center for Molecular Biomedicine, Aviano, Pordenone, Italy;; c Department of Molecular Sciences and Nanosystems, Ca’ Foscari Università di Venezia, Venezia-Mestre, Italy;; d Sbarro Institute for Cancer Research and Molecular Medicine, Center for Biotechnology, College of Science and Technology, Temple University, Philadelphia, PA, USA

**Keywords:** Monoacylglycerol lipase inhibitors, endocannabinoids, docking, molecular dynamic simulations

## Abstract

Monoacylglycerol lipase is a serine hydrolase that plays a major role in the degradation of the endocannabinoid neurotransmitter 2-arachidonoylglycerol. A wide number of MAGL inhibitors are reported in literature; however, many of them are characterised by an irreversible mechanism of action and this behavior determines an unwanted chronic MAGL inactivation, which acquires a functional antagonism of the endocannabinoid system. The possible use of reversible MAGL inhibitors has only recently been explored, due to the lack of known compounds possessing efficient reversible inhibitory activities. In this work, we report a new series of terphenyl-2-methyloxazol-5(4*H*)-one derivatives characterised by a reversible MAGL-inhibition mechanism. Among them, compound **20b** showed to be a potent MAGL reversible inhibitor (IC_50_ = 348 nM) with a good MAGL/FAAH selectivity. Furthermore, this compound showed antiproliferative activities against two different cancer cell lines that overexpress MAGL.

## Introduction

**Figure 1.**
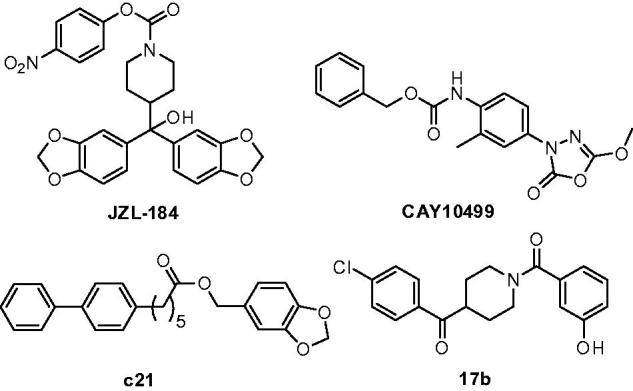
Structures of some of the most relevant MAGL inhibitors.

Endocannabinoids are lipid transmitters that act as endogenous ligands of the CB_1_ and CB_2_ cannabinoid receptors. The endogenous ligands 2-arachidonoylglycerol (2-AG) and *N*-arachidonoyl ethanolamine (AEA) are considered as the two major endocannabinoids and modulate multiple physiological processes including pain, inflammation, appetite, memory and emotion^1^. Their signaling activity is terminated by enzymatic hydrolysis, which is mainly mediated by serine hydrolase monoacylglicerol lipase (MAGL) and fatty acid amide hydrolase (FAAH), respectively^2^. Because of its key role in 2-AG catabolism, selective inactivation of MAGL represents an interesting approach for obtaining the desirable effects of indirect cannabinoid receptors activation. MAGL inhibition in the periphery produces CB_1_-dependent antinociceptive effects in mouse models of noxious chemical, inflammatory, thermal and neuropathic pain^3^. Pharmacological and genetic blockades of MAGL exhibit anti-inflammatory effects in the brain and neuroprotective effects in mouse models of Parkinson’s and Alzheimer’s disease^4^. Other studies suggest that MAGL inhibition produces anti-anxiety responses^5^ and could be useful for modulating drug dependence of opiates^6^. Finally, MAGL is upregulated in aggressive cancer cells and primary tumors and its inhibition in aggressive breast, ovarian, and melanoma cancer cells impairs cell migration, invasiveness, and tumorigenicity^7^. Over the past 10 years, great efforts have been done for identifying novel MAGL inhibitors^8–13^; however, almost all the reported compounds are characterised by an irreversible MAGL-inhibition mechanism^2^. Among this wide class of MAGL inhibitors, **JZL184**^11^ and **CAY10499**^8^ (Figure 1) are the two main compounds that are used as reference inhibitors for most of the cellular and animal experiments in which MAGL is studied. However, as reported by Scholsburg et al.^14^, the repeated administrations in mice of an irreversible MAGL inhibitor produces cross-tolerance to CB_1_ agonists. Furthermore, chronic MAGL blockade causes impaired endocannabinoid-dependent synaptic plasticity, physical dependence and desensitised brain CB_1_ receptors^14^. Considering all these negative effects associated to the irreversible MAGL inhibition, the need to discover selective and reversible MAGL inhibitors constitutes a challenging opportunity to target MAGL with minimal occurrence of unwanted side effects. To our knowledge, only few compounds described as good reversible MAGL inhibitors have so far been reported in literature. In 2014, a reversible MAGL inhibitor (compound **c21**, Figure 1) was tested *in vivo* using the EAE (experimental allergic encephalomyelitis) mouse model and the ligand ameliorated the clinical progression of the multiple sclerosis mouse model. Very importantly, the therapeutic effects were not accompanied by catalepsy or other motor impairments which have been instead observed after the administration of irreversible MAGL inhibitors^15^. Finally, in 2016, Tuccinardi et al.^16^ reported a novel class of benzoylpiperidine derivatives as potent and selective MAGL reversible inhibitors possessing antiproliferative activity against ovarian cancer cell lines (Figure 1, compound **17b**)^17^.

**Figure 2.**
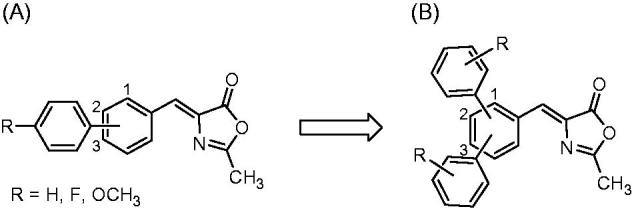
Structural evolution of methyleneoxazol-5(4*H*)-one scaffold: previously developed biphenyl 2-methyloxazol-5(4*H*)-one compounds (A) and newly synthesised terphenyl-2-methyloxazol-5(4*H*)-one derivatives (B).

Very recently, the same authors developed a class of biphenyl 2-methyloxazol-5(4*H*)-one compounds of general structure **A** (Figure 2) that inhibits MAGL reversibly and selectively. Modeling studies revealed that the pharmacophoric portions are both the 2-methyloxazol-5(4*H*)-one ring, interacting with the catalytic S122 of the enzyme, and the peripheral phenyl ring, which nicely fits into a lipophilic cavity of the protein^18^. On the basis of these results, and considering the wide available space within the MAGL lipophilic cavity, we decided to add a second aromatic portion, in order to adequately fill this region, while maintaining the same central scaffold of the previous series of compounds (general structure **B**, Figure 2). Therefore, we initially designed and synthesised terphenyl-2-methyloxazol-5(4*H*)-one derivatives displaying all the possible combinations of substitution positions on the central phenyl ring by inserting unsubstituted phenyl rings. In a second time, after a preliminary evaluation, the best obtained regioisomeric compound in terms of inhibitory enzymatic activity was selected for further fine chemical modifications and, therefore, variously substituted phenyl rings were introduced in the selected positions to determine which kind of group was most suitable to improve MAGL inhibitory potency and enzyme selectivity properties.

## Material and methods

### Chemistry

Commercially available chemicals were purchased from Sigma-Aldrich (St. Louis, MO, USA) or Alfa Aesar-Thermo Fisher Scientific (Karlsruhe, Germany) and used without further purification. **JZL-184** and **CAY10499** were purchased from Cayman Chemical. NMR spectra were obtained with a Bruker Avance III 400 MHz spectrometer. Chemical shifts (δ) are reported in parts per million downfield from tetramethylsilane and referenced from solvent references. HPLC analysis: all target compounds (i.e. assessed in biological assays) were ≥94% pure by HPLC, confirmed via UV detection (λ = 310 nm). Analytical reversed-phase HPLC was conducted using a Kinetex EVO C18 column (5 µm, 150 × 4.6 mm, Phenomenex, Inc.); eluent A, water; eluent B, CH_3_CN; after 3 min at 25% B, a gradient was formed from 25 to 85% of B in 4 min and held at 85% of B for 8 min; flow rate was 1 ml/min. Chromatographic separations were performed on silica gel columns by flash chromatography (Kieselgel 60, 0.040–0.063 mm; Merck). Reactions were followed by thin-layer chromatography (TLC) on Aldrich aluminum silica gel (F254) sheets that were visualised under a UV lamp. Evaporation was performed *in vacuo* (rotating evaporator). Sodium sulfate was always used as the drying agent. Elemental analysis has been used to determine the purity of target compounds. Analytical results are within ±0.40% of the theoretical values.

#### General procedure for the formation of terphenyl derivatives 6, 12, 14 and 19a–h

A solution of Pd(OAc)_2_ (0.06 eq) and triphenylphosphine (0.30 eq) in absolute ethanol (6 ml/2.7 mmol halogenated derivative) and toluene (6 ml/2.7 mmol halogenated derivative) was stirred at room temperature (RT) under nitrogen for 10 min. After that period, commercially available dibromo- or dichloro-substituted aldehydes **2**, **10** or **11** (1 eq), 2 M aqueous Na_2_CO_3_ (6 ml/2.7 mmol halogenated derivative), and opportunely substituted phenylboronic acid (3.2 eq) were sequentially added. The resulting mixture was heated at 100 °C in a sealed vial under nitrogen for 24 h. After being cooled to RT, it was checked by TLC and if starting material was still present or it was visible the presence of two close spots (probable mono- and di-substitution products), it was added Pd(OAc)_2_ (0.03 eq), triphenylphosphine (0.15 eq) and phenylboronic acid (1.6 eq). The mixture was heated again at 100 °C for further 24 h. Finally, the mixture was cooled to RT, diluted with water and extracted with EtOAc. The combined organic phase was dried and concentrated. The crude product was purified by flash chromatography using the indicated eluent and pure fractions containing the desired compound were evaporated to dryness affording the desired product.

#### (1,1′:3′,1′′-Terphenyl)-4′-carbaldehyde (6)

Yellow crystalline solid, yield: 94% (277.4 mg) from **2** and phenylboronic acid. *R_f_* = 0.11 (*n*-hexane/EtOAc 98:2). ^1^H-NMR (CDCl_3_, 400 MHz) δ (ppm): 7.39–7.53 (m, 8H), 7.65–7.70 (m, 3H), 7.73 (dd, 1H, *J* = 8.2, 1.0 Hz), 8.12 (d, 1H, *J* = 8.0 Hz), 10.02 (s, 1H).

#### (1,1′:3′,1′′-Terphenyl)-5′-carbaldehyde (12)

White solid, yield: 93% (274.0 mg) from **10** and phenylboronic acid. *R_f_* = 0.08 (*n*-hexane/EtOAc 98:2). ^1^H-NMR (CDCl_3_, 400 MHz) δ (ppm): 7.43 (tt, 2H, *J* = 7.4, 1.7 Hz), 7.48–7.53 (m, 4H), 7.67–7.71 (m, 4H), 8.06–8.10 (m, 3H), 10.16 (s, 1H).

#### (1,1′:4′,1′′-Terphenyl)-2′-carbaldehyde (14)

White solid, yield: 80% (236.0 mg) from **11** and phenylboronic acid. *R_f_* = 0.17 (*n*-hexane/EtOAc 98:2). ^1^H-NMR (CDCl_3_, 400 MHz) δ (ppm): 7.38–7.57 (m, 9H), 7.67–7.71 (m, 2H), 7.89 (dd, 1H, *J* = 8.0, 2.1 Hz), 8.28 (d, 1H, *J* = 2.0 Hz), 10.05 (s, 1H).

#### 4,4′′-Difluoro-(1,1′:4′,1′′-terphenyl)-2′-carbaldehyde (19a)

White solid, yield: 97% (325.5 mg) from **11** and 4-fluorophenylboronic acid. *R_f_* = 0.18 (*n*-hexane/EtOAc 98:2). ^1^H-NMR (CDCl_3_, 400 MHz) δ (ppm): 7.14–7.22 (m, 4H), 7.39 (double AA′XX′, 2H, ^4^*J*_HF-m_= 5.3 Hz, *J*_AX_ = 8.8 Hz, *J*_AA′/XX′_ = 2.5 Hz), 7.50 (d, 1H, *J* = 7.9 Hz), 7.64 (double AA′XX′, 2H, ^4^*J*_HF-m_ = 5.2 Hz, *J*_AX_ = 8.8 Hz, *J*_AA′/XX′_ = 2.6 Hz), 7.83 (dd, 1H, *J* = 8.0, 2.1 Hz), 8.20 (d, 1H, *J* = 2.0 Hz), 10.02 (s, 1H).

#### 4,4′′-Dimethoxy-(1,1′:4′,1′′-terphenyl)-2′-carbaldehyde (19b)

White solid, yield: 99% (363.5 mg) from **11** and 4-methoxyphenylboronic acid. *R_f_* = 0.25 (*n*-hexane/EtOAc 9:1). ^1^H-NMR (CDCl_3_, 400 MHz) δ (ppm): 3.87 (s, 3H), 3.89 (s, 3H), 6.99–7.05 (m, 4H), 7.35 (AA′XX′, 2H, *J*_AX_ = 8.8 Hz, *J*_AA′/XX′_ = 2.1 Hz), 7.49 (d, 1H, *J* = 8.0 Hz), 7.62 (AA′XX′, 2H, *J*_AX_ = 8.9 Hz, J_AA′/XX′_ = 2.2 Hz), 7.82 (dd, 1H, *J* = 8.0, 2.1 Hz), 8.20 (d, 1H, *J* = 2.0 Hz), 10.05 (s, 1H).

#### 4,4′′-Bis(trifluoromethoxy)-(1,1′:4′,1′′-terphenyl)-2′-carbaldehyde (19c)

Colorless oil, yield: 89% (432.1 mg) from **11** and 4-trifluoromethoxyphenylboronic acid. *R_f_* = 0.13 (*n*-hexane/EtOAc 99:1). ^1^H-NMR (CDCl_3_, 400 MHz) δ (ppm): 7.32–7.38 (m, 4H), 7.46 (AA′XX′, 2H, *J*_AX_ = 8.8 Hz, *J*_AA′/XX′_ = 2.4 Hz), 7.52 (d, 1H, *J* = 8.0 Hz), 7.69 (AA′XX′, 2H, *J*_AX_ = 8.9 Hz, *J*_AA′/XX′_ = 2.5 Hz), 7.86 (dd, 1H, *J* = 8.0, 2.2 Hz), 8.23 (d, 1H, *J* = 1.8 Hz), 10.03 (s, 1H).

#### 4,4′′-Bis(trifluoromethyl)-(1,1′:4′,1′′-terphenyl)-2′-carbaldehyde (19d)

White solid, yield: 95% (424.8 mg) from **11** and 4-trifluoromethylphenylboronic acid. *R_f_* = 0.15 (*n*-hexane/EtOAc 99:1). ^1^H-NMR (CDCl_3_, 400 MHz) δ (ppm): 7.54–7.59 (m, 3H), 7.74–7.81 (m, 6H), 7.92 (dd, 1H, *J* = 8.0, 2.1 Hz), 8.30 (d, 1H, *J* = 1.8 Hz), 10.03 (s, 1H).

#### 3,3′′-Difluoro-(1,1′:4′,1′′-terphenyl)-2′-carbaldehyde (19e)

White solid, yield: 94% (316.0 mg) from **11** and 3-fluorophenylboronic acid. *R_f_* = 0.10 (*n*-hexane/EtOAc 98:2). ^1^H-NMR (CDCl_3_, 400 MHz) δ (ppm): 7.07–7.14 (m, 1H), 7.14–7.21 (m, 3H), 7.35–7.40 (m, 1H), 7.42–7.50 (m, 3H), 7.53 (d, 1H, *J* = 8.0 Hz), 7.87 (dd, 1H, *J* = 8.0, 2.1 Hz), 8.25 (d, 1H, *J* = 2.0 Hz), 10.04 (s, 1H).

#### 2,5-Bis[benzo(d)(1,3)dioxol-5-yl]benzaldehyde (19f)

Dark yellow solid, yield: 75% (295.6 mg) from **11** and 3,4-(methylenedioxy)phenylboronic acid. *R_f_* = 0.18 (*n*-hexane/EtOAc 9:1). ^1^H-NMR (CDCl_3_, 400 MHz) δ (ppm): 6.03 (s, 2H), 6.06 (s, 2H), 6.83 (dd, 1H, *J* = 7.8, 1.9 Hz), 6.89–6.94 (m, 3H), 7.12–7.16 (m, 2H), 7,47 (d, 1H, *J* = 8.0 Hz), 7,77 (dd, 1H, *J* = 8.0, 2.1 Hz), 8.15 (d, 1H, *J* = 2.0 Hz), 10.05 (s, 1H).

#### 3,3′′-Difluoro-4,4′′-dimethoxy-(1,1′:4′,1′′-terphenyl)-2′-carbaldehyde (19g)

Pearly white solid, yield: 82% (329.5 mg) from **11** and 3-fluoro-4-methoxyphenylboronic acid. *R_f_* = 0.13 (*n*-hexane/EtOAc 9:1). ^1^H-NMR (CDCl_3_, 400 MHz) δ (ppm): 3.96 (s, 3H), 3.97 (s, 3H), 7.04–7.12 (m, 3H), 7.19 (dd, 1H, *J* = 11.6, 2.0 Hz), 7.38–7.44 (m, 2H), 7.48 (d, 1H, *J* = 8.0 Hz), 7.80 (dd, 1H, *J* = 8.0, 2.1 Hz), 8.18 (d, 1H, *J* = 2.0 Hz), 10.05 (s, 1H).

#### 4,4′′-Dichloro-(1,1′:4′,1′′-terphenyl)-2′-carbaldehyde (19h)

White solid, yield: 61% (143.0 mg) from **11** and 4-chlorophenylboronic acid. *R_f_* = 0.40 (*n*-hexane/EtOAc 98:2). ^1^H-NMR (CDCl_3_, 400 MHz) δ (ppm): 7.36 (AA′XX′, 2H, *J*_AX_ = 8.6 Hz, *J*_AA′/XX′_ = 2.3 Hz), 7.44–7.52 (m, 5H), 7.61 (AA′XX′, 2H, *J*_AX_ = 8.7 Hz, *J*_AA′/XX′_ = 2.3 Hz), 7.85 (dd, 1H, *J* = 8.0, 2.1 Hz), 8.22 (d, 1H, *J* = 2.1 Hz), 10.03 (s, 1H).

#### Procedure for the synthesis of (1,1′:2′,1′′-terphenyl)-4′-carbaldehyde (4)

3,4-Dichlorobenzaldehyde **1** (500 mg, 2.86 mmol, 1 eq) was placed in a vial together with phenylboronic acid (1.39 g, 11.4 mmol), potassium phosphate (2.67 g, 12.6 mmol), Pd(OAc)_2_ (57.8 mg, 0.0858 mmol), tetrabutylammonium bromide (TBAB) (14.3 g, 44.3 mmol) and water (6.4 ml). The vial was sealed and heated under stirring at 125 °C for 48 h. The reaction mixture was cooled to RT and then diluted with water. The water phase was acidified with 1 N aqueous HCl and repeatedly extracted with EtOAc. The combined organic phase was washed with brine, dried over anhydrous sodium sulfate and evaporated to afford a crude residue that was purified by column chromatography over silica gel using *n*-hexane/EtOAc 98:2 (*R_f_* = 0.16) as the eluent, to give pure **4** as a yellow oily compound (157.9 mg, 21% yield). ^1^H-NMR (DMSO-d_6_, 400 MHz) δ (ppm): 7.14–7.20 (m, 4H), 7.25–7.32 (m, 6H), 7,65 (d, 1H, *J* = 8.0 Hz), 7.94 (d, 1H, *J* = 1.2 Hz), 7.98 (dd, 1H, *J* = 7.6, 1.6 Hz), 10.11 (s, 1H).

#### Procedure for the synthesis of (1,1′:2′,1′′-terphenyl)-3′-carbaldehyde (8)

A solution of Pd(OAc)_2_ (0.06 eq) and triphenylphosphine (0.30 eq) in absolute ethanol (6 ml/2.7 mmol halogenated derivative) and toluene (6 ml/2.7 mmol halogenated derivative) was stirred at RT under nitrogen for 10 min. After that period, commercially available 2,3-dichlorobenzaldehyde **3** (300 mg, 1.71 mmol, 1 eq), 2 M aqueous Na_2_CO_3_ (6 ml/2.7 mmol halogenated derivative), and phenylboronic acid (3.2 eq) were sequentially added. The resulting mixture was heated at 100 °C in a sealed vial under nitrogen for 24 h. After being cooled to RT, the mixture was diluted with water and extracted with EtOAc. The combined organic phase was dried and concentrated. Since ^1^H-NMR analysis revealed the formation of a mono-substitution product, the crude (320 mg) was dissolved in anhydrous dioxane (3.7 ml) and treated, under nitrogen, with cesium carbonate (1.7 eq), phenylboronic acid (1.6 eq), Pd_2_(dba)_3_ (0.032 eq), and a 20% solution of tricyclohexylphosphine in toluene (0.08 eq). The reaction was heated at 100 °C in a sealed vial overnight. The reaction mixture was then cooled to RT, diluted with EtOAc, and filtered through a Celite pad. The organic filtrate was concentrated under vacuum, and the crude product was purified by flash chromatography (*n*-hexane/EtOAc 99:1, *R_f_* = 0.08) to yield pure **8** as a white solid (244.3 mg, 55% total yield over two steps). ^1^H-NMR (CDCl_3_, 400 MHz) δ (ppm): 7.03–7.07 (m, 2H), 7.08–7.13 (m, 2H), 7.15–7.20 (m, 3H), 7.23–7.29 (m, 3H), 7.56 (t, 1H, *J* = 7.7 Hz), 7.65 (dd, 1H, *J* = 7.6, 1.5 Hz), 8.04 (dd, 1H, *J* = 7.8, 1.5 Hz), 9.81 (s, 1H).

#### Procedure for the synthesis of (1,1′:3′,1′′-terphenyl)-2′-carbaldehyde (17)

2,6-Dichlorobenzaldehyde **16** (500 mg, 2.86 mmol, 1 eq) was dissolved in anhydrous dioxane (7.2 ml) and treated, under nitrogen, with cesium carbonate (3.4 eq), phenylboronic acid (3.2 eq), Pd_2_(dba)_3_ (0.064 eq), and a 20% solution of tricyclohexylphosphine in toluene (0.16 eq). The reaction was heated at 100 °C in a sealed vial overnight. After being cooled to RT, it was added Pd_2_(dba)_3_ (0.032 eq), tricyclohexylphosphine (0.08 eq) and phenylboronic acid (1.6 eq). The mixture was heated again at 100 °C for further 24 h. The reaction mixture was then cooled to RT, diluted with EtOAc, and filtered through a Celite pad. The organic filtrate was concentrated under vacuum, and the crude product was purified by flash chromatography (*n*-hexane/EtOAc 98:2, *R_f_* = 0.13) to yield pure **17** as a crystalline yellow solid (685 mg, 93% yield). ^1^H-NMR (CDCl_3_, 400 MHz) δ (ppm): 7.33–7.47 (m, 12H), 7.60 (dd, 1H, *J* = 7.9, 7.4 Hz), 9.96 (s, 1H).

#### General procedure for the preparation of the 2-methyloxazol-5(4H)-one derivatives 5, 7, 9, 13, 15

A mixture of diphenyl-substituted benzaldehydes **4**, **6**, **8**, **12** or **14** (1 eq), *N*-acetylglycine (1 eq) and sodium acetate (1 eq) in acetic anhydride (5 ml/5 mmol aldehyde) was stirred at reflux for 5 h and then warmed to RT. The reaction was quenched with water and extracted with AcOEt. The organic layer was washed sequentially with water and saturated brine, dried over Na_2_SO_4_ and the solvent was removed under reduced pressure. The residue was purified with a flash column chromatography using the indicated eluent and pure fractions containing the desired compound were evaporated to dryness affording the desired product.

#### (Z)-4-[(1,1′:2′,1′′-Terphenyl)-4′-ylmethylene]-2-methyloxazol-5(4H)-one (5)

Yellow solid, yield: 23% (44.4 mg) from **4**. *R_f_* = 0.21 (*n*-hexane/EtOAc 95:5). ^1^H-NMR (CDCl_3_, 400 MHz) δ (ppm): 2.41 (s, 3H), 7.12–7.18 (m, 4H), 7.20–7.27 (m, 7H), 7.52 (d, 1H, *J* = 8.0 Hz), 8.07 (d, 1H, *J* = 1.8 Hz), 8.19 (dd, 1H, *J* = 8.2, 1.7 Hz). 13C-NMR (CDCl_3_, 100 MHz) δ (ppm): 15.86, 127.01, 127.22, 128.16 (2 C), 128.19 (2 C), 129.86 (2 C), 129.99 (2 C), 131.09, 131.14, 131.36, 132.54, 132.96, 134.56, 140.75, 140.86, 141.34, 143.50, 166.29, 167.98. HPLC analysis: retention time = 11.207 min; peak area, 95%. Elemental analysis for C_23_H_17_NO_2_ calculated: % C, 81.40; % H, 5.05; % N, 4.13; found: % C, 81.10; % H, 5.03; % N, 4.12.

#### (Z)-4-[(1,1′:3′,1′′-Terphenyl)-4′-ylmethylene]-2-methyloxazol-5(4H)-one (7)

Yellow solid, yield: 11% (55.2 mg) from **6**. *R_f_* = 0.13 (*n*-hexane/EtOAc 95:5). ^1^H-NMR (CDCl_3_, 400 MHz) δ (ppm): 2.43 (s, 3H), 7.24 (s, 1H), 7.35–7.50 (m, 8H), 7.63–7.69 (m, 3H), 7.72 (dd, 1H, *J* = 8.3, 1.9 Hz), 8.79 (d, 1H, *J* = 8.3 Hz). 13C-NMR (CDCl_3_, 100 MHz) δ (ppm): 15.89, 126.48, 127.29, 127.35, 128.19, 128.30, 128.62, 128.69 (2 C), 129.05, 129.11 (2 C), 129.15, 130.06, 130.09, 132.70, 132.71, 139.95, 140.09, 143.40, 145.72, 166.25, 167.75. HPLC analysis: retention time = 11.349 min; peak area, 96%. Elemental analysis for C_23_H_17_NO_2_ calculated: % C, 81.40; % H, 5.05; % N, 4.13; found: % C, 81.32; % H, 5.04; % N, 4.12.

#### (Z)-4-[(1,1′:2′,1′′-Terphenyl)-3′-ylmethylene]-2-methyloxazol-5(4H)-one (9)

Yellow solid, yield: 13% (39.4 mg) from **8**. *R_f_* = 0.14 (*n*-hexane/EtOAc 95:5). ^1^H-NMR (CDCl_3_, 400 MHz) δ (ppm): 2.41 (s, 3H), 6.90–7.05 (m, 5H), 7.12–7.17 (m, 3H), 7.20–7.25 (m, 3H), 7.48 (dd, 1H, *J* = 7.6, 1.5 Hz), 7.54 (t, 1H, *J* = 7.7 Hz), 8.65 (dd, 1H, *J* = 7.8, 1.4 Hz). 13C-NMR (CDCl_3_, 100 MHz) δ (ppm): 15.86, 126.64, 127.57, 127.81 (3 C), 128.01 (2 C), 129.82 (2 C), 130.97, 131.03, 131.29 (2 C), 132.23, 132.74, 132.87, 138.12, 141.21, 142.40, 143.29, 166.41, 167.56. HPLC analysis: retention time = 10.983 min; peak area, 94%. Elemental analysis for C_23_H_17_NO_2_ calculated: % C, 81.40; % H, 5.05; % N, 4.13; found: % C, 81.07; % H, 5.03; % N, 4.11.

#### (Z)-4-[(1,1′:3′,1′′-Terphenyl)-5′-ylmethylene]-2-methyloxazol-5(4H)-one (13)

Yellow solid, yield: 41% (142.0 mg) from **12**. *R_f_* = 0.17 (*n*-hexane/EtOAc 95:5). ^1^H-NMR (CDCl_3_, 400 MHz) δ (ppm): 2.43 (s, 3H), 7.27 (s, 1H), 7.41 (tt, 2H, *J* = 7.3, 1.5 Hz), 7.47–7.53 (m, 4H), 7.66–7.70 (m, 4H), 7.86 (t, 1H, *J* = 1.7 Hz), 8.30 (d, 2H, *J* = 1.6 Hz). 13C-NMR (CDCl_3_, 100 MHz) δ (ppm): 15.73, 128.06, 128.76, 128.84, 129.89 (11 C), 130.36, 130.58, 134.59, 135.60, 141.13, 143.16, 168.14, 168.21. HPLC analysis: retention time = 11.496 min; peak area, 97%. Elemental analysis for C_23_H_17_NO_2_ calculated: % C, 81.40; % H, 5.05; % N, 4.13; found: % C, 81.68; % H, 5.06; % N, 4.14.

#### (Z)-4-[(1,1′:4′,1′′-Terphenyl)-2′-ylmethylene]-2-methyloxazol-5(4H)-one (15)

Yellow solid, yield: 15% (43.0 mg) from **14**. *R_f_* = 0.18 (*n*-hexane/EtOAc 95:5). ^1^H-NMR (CDCl_3_, 400 MHz) δ (ppm): 2.42 (s, 3H), 7.11 (s, 1H), 7.41–7.57 (m, 9H), 7.75–7.79 (m, 2H), 7.84 (dd, 1H, *J* = 8.0, 2.0 Hz), 9.10 (d, 1H, *J* = 2.0 Hz). 13C-NMR (CDCl_3_, 100 MHz) δ (ppm): 15.74, 127.82 (3 C), 128.67, 128.70, 128.86, 129.44 (2 C), 129.92, 129.97 (2 C), 130.80 (2 C), 131.16, 131.80, 132.19, 134.52, 140.52, 141.24, 144.54, 168.16, 168.23. HPLC analysis: retention time = 11.425 min; peak area, 95%. Elemental analysis for C_23_H_17_NO_2_ calculated: % C, 81.40; % H, 5.05; % N, 4.13; found: % C, 81.65; % H, 5.06; % N, 4.15.

#### General procedure for the preparation of the 2-methyloxazol-5(4H)-one derivatives 20a–h

The procedure for the synthesis of these compounds is similar to that used for previous analog final products, with the exception of the used equivalents of *N*-acetylglycine (2 eq) and sodium acetate (2 eq).

#### (Z)-4-{[4,4′′-Difluoro-(1,1′:4′,1′′-terphenyl)-2′-yl]methylene}-2-methyloxazol-5(4H)-one (20a)

Yellow solid, yield: 35% (46.5 mg) from **19a**. *R_f_* = 0.20 (*n*-hexane/EtOAc 95:5). ^1^H-NMR (acetone-d_6_, 400 MHz) δ (ppm): 2.43 (s, 3H), 7.06 (s, 1H), 7.28–7.34 (m, 4H), 7.48 (double AA′XX′, 2H, 4JHF_-m_= 5.4 Hz, *J*_AX_ = 8.9 Hz, *J*_AA′/XX′_ = 2.2 Hz), 7.55 (d, 1H, *J* = 8.0 Hz), 7.78–7.84 (m, 3H), 9.04 (d, 1H, *J* = 1.9 Hz). 13C-NMR (acetone-d_6_, 100 MHz) δ (ppm): 15.71, 116.25 (d, 2 C, *J* = 21.4 Hz), 116.69 (d, 2 C, *J* = 21.8 Hz), 128.23, 129.75 (d, 2 C, *J* = 8.1 Hz), 129.81, 131.03, 131.86, 132.32, 132.74 (d, 2 C, *J* = 8.1 Hz), 134.75, 136.68 (d, *J* = 3.5 Hz), 137.27 (d, *J* = 3.2 Hz), 140.29, 143.29, 163.60 (d, *J* = 234.1 Hz),163.60 (d, *J* = 257.3 Hz), 168.07, 168.42. HPLC analysis: retention time = 11.375 min; peak area, 96%. Elemental analysis for C_23_H_15_F_2_NO_2_ calculated: % C, 73.59; % H, 4.03; % N, 3.73; found: % C, 73.35; % H, 4.01; % N, 3.72.

#### (Z)-4-{[4,4′′-Dimethoxy-(1,1′:4′,1′′-terphenyl)-2′-yl]methylene}-2-methyloxazol-5(4H)-one (20b)

Yellow solid, yield: 19% (23.8 mg) from **19b**. *R_f_* = 0.15 (*n*-hexane/EtOAc 9:1). ^1^H-NMR (acetone-d_6_, 400 MHz) δ (ppm): 2.42 (s, 3H), 3.88 (s, 3H), 3.89 (s, 3H), 7.06–7.11 (m, 4H), 7.14 (s, 1H), 7.35 (AA′XX′, 2H, *J*_AX_ = 8.8 Hz, *J*_AA′/XX′_ = 2.6 Hz), 7.49 (d, 1H, *J* = 7.8 Hz), 7.70 (AA′XX′,2H, *J*_AX_ = 8.9 Hz, *J*_AA′/XX′_ = 2.6 Hz), 7.77 (dd, 1H, *J* = 8.1, 2.1 Hz), 9.03 (d, 1H, *J* = 2.0 Hz). 13C-NMR (acetone-d_6_, 100 MHz) δ (ppm): 15.71, 55.60, 55.65, 114.55 (2 C), 114.85, 115.27 (2 C), 115.36, 128.67 (2 C), 128.82, 129.46, 130.62, 131.70, 131.84 (2 C), 131.99, 132.10, 139.79, 140.48, 160.38, 160.51, 166.44, 168.25. HPLC analysis: retention time = 11.155 min; peak area, 95%. Elemental analysis for C_25_H_21_NO_4_ calculated: % C, 75.17; % H, 5.30; % N, 3.51; found: % C, 74.90; % H, 5.28; % N, 3.50.

#### (Z)-4-{[4,4′′-Bis(trifluoromethoxy)-(1,1′:4′,1′′-terphenyl)-2′-yl]methylene}-2-methyloxazol-5(4H)-one (20c)

Yellow solid, yield: 32% (161.3 mg) from **19c**. *R_f_* = 0.08 (*n*-hexane/EtOAc 98:2). ^1^H-NMR (acetone-d_6_, 400 MHz) δ (ppm): 2.43 (s, 3H), 7.06 (s, 1H), 7.48–7.53 (m, 4H), 7.57–7.62 (m, 3H), 7.86–7.90 (m, 1H), 7.91 (AA′XX′, 2H, *J*_AX_ = 8.6 Hz, *J*_AA′/XX′_ = 1.9 Hz), 9.09 (d, 1H, *J* = 1.8 Hz). 13C-NMR (acetone-d_6_, 100 MHz) δ (ppm): 15.74, 121.54 (q, 2 C, *J* = 256.0 Hz), 121.94, 122.51, 127.87, 129.64 (2 C), 129.97 (2 C), 131.29, 131.99 (2 C), 132.42, 132.64 (2 C), 135.07, 139.52, 140.04, 140.15, 143.19, 149.83, 149.84, 168.00, 168.66. HPLC analysis: retention time = 13.106 min; peak area, 94%. Elemental analysis for C_25_H_15_F_6_NO_4_ calculated: % C, 59.18; % H, 2.98; % N, 2.76; found: % C, 59.40; % H, 2.99; % N, 2.77.

#### (Z)-4-{[4,4′′-Bis(trifluoromethyl)-(1,1′:4′,1′′-terphenyl)-2′-yl]methylene}-2-methyloxazol-5(4H)-one (20d)

Yellow solid, yield: 54% (260.3 mg) from **19d**. *R_f_* = 0.18 (*n*-hexane/EtOAc 9:1). ^1^H-NMR (acetone-d_6_, 400 MHz) δ (ppm): 2.44 (s, 3H), 7.04 (s, 1H), 7.65 (d, 1H, *J* = 8.0 Hz), 7.68–7.73 (m, 2H), 7.87–7.93 (m, 4H), 7.96 (dd, 1H, *J* = 8.1, 2.0 Hz), 7.99–8.04 (m, 2H), 9.16 (d, 1H, *J* = 2.0 Hz). 13C-NMR (acetone-d_6_, 100 MHz) δ (ppm): 15.75, 125.36 (q, *J* = 271.5 Hz), 125.45 (q, *J* = 271.2 Hz), 126.37 (q, 2 C, *J* = 3.7 Hz), 126.91 (q, 2 C, *J* = 3.9 Hz), 127.46, 128.60, 130.14, 130.27 (q, *J* = 32.3 Hz), 130.48 (q, *J* = 32.3 Hz), 131.57 (2 C), 132.02, 132.51, 135.34, 140.33, 143.55, 144.41 (q, 2 C, *J* = 1.4 Hz), 144.70 (q, 2 C, *J* = 1.4 Hz), 167.93, 168.87. HPLC analysis: retention time = 12.638 min; peak area, 97%. Elemental analysis for C_25_H_15_F_6_NO_2_ calculated: % C, 63.16; % H, 3.18; % N, 2.95; found: % C, 63.38; % H, 3.17; % N, 2.96.

#### (Z)-4-{[3,3′′-Difluoro-(1,1′:4′,1′′-terphenyl)-2′-yl]methylene}-2-methyloxazol-5(4H)-one (20e)

Yellow solid, yield: 45% (89.5 mg) from **19e**. *R_f_* = 0.20 (*n*-hexane/EtOAc 95:5). ^1^H-NMR (acetone-d_6_, 400 MHz) δ (ppm): 2.43 (s, 3H), 7.05 (s, 1H), 7.18–7.30 (m, 4H), 7.48–7.63 (m, 5H), 7.87 (dd, 1H, *J* = 8.0, 2.0 Hz), 9.07 (d, 1H, *J* = 2.0 Hz). 13C-NMR (acetone-d_6_, 100 MHz) δ (ppm): 15.75, 114.48 (d, *J* = 22.8 Hz), 115.44 (d, *J* = 21.3 Hz), 115.70 (d, *J* = 21.3 Hz), 117.47 (d, *J* = 22.0 Hz), 123.80 (d, *J* = 2.9 Hz), 126.95 (d, *J* = 2.9 Hz), 127.83, 129.90, 131.22, 131.35 (d, *J* = 8.1 Hz) 131.88 (d, *J* = 9.9 Hz), 132.34, 135.02, 140.22, 142.73 (d, *J* = 7.9 Hz), 143.29 (d, *J* = 7.3 Hz), 143.45, 143.47, 163.56 (d, *J* = 245.7 Hz), 164.21 (d, *J* = 244.3 Hz), 168.02, 168.63. HPLC analysis: retention time = 11.405 min; peak area, 97%. Elemental analysis for C_23_H_15_F_2_NO_2_ calculated: % C, 73.59; % H, 4.03; % N, 3.73; found: % C, 73.40; % H, 4.02; % N, 3.72.

#### (Z)-4–[2,5-Bis(benzo[d][1,3]dioxol-5-yl)benzylidene]-2-methyloxazol-5(4H)-one (20f)

Yellow solid, yield: 11% (25.9 mg) from **19f**. *R_f_* = 0.10 (*n*-hexane/Et_2_O 9:1). ^1^H-NMR (acetone-d_6_, 400 MHz) δ (ppm): 2.42 (s, 3H), 6.09 (s, 2H), 6.11 (s, 2H), 6.86 (dd, 1H, *J* = 8.0, 1.8 Hz), 6.93 (d, 1H, *J* = 1.6 Hz), 6.97–7.01 (m, 2H), 7.14 (s, 1H), 7.22–7.27 (m, 2H), 7.48 (d, 1H, *J* = 8.0 Hz), 7.74 (dd, 1H, *J* = 8.1, 2.1 Hz), 8.97 (d, 1H, *J* = 2.0 Hz). 13C-NMR (acetone-d_6_, 100 MHz) δ (ppm): 15.73, 66.82, 66.93, 102.39, 102.49, 108.02, 109.04, 109.55, 110.91, 121.47, 124.64, 128.92, 129.63, 130.82, 131.71, 132.22, 134.31, 135.23, 140.74, 143.81, 148.61, 148.93, 149.48. 168.08, 168.22. HPLC analysis: retention time = 10.849 min; peak area, 95%. Elemental analysis for C_25_H_17_NO_6_ calculated: % C, 70.25; % H, 4.01; % N, 3.28; found: % C, 69.98; % H, 3.99; % N, 3.27.

#### (Z)-4-{[3,3′′-Difluoro-4,4′′-dimethoxy-(1,1′:4′,1′′-terphenyl)-2′-yl]methylene}-2-methyloxazol-5(4H)-one (20g)

Yellow solid, yield: 12% (49.2 mg) from **19g**. *R_f_* = 0.13 (*n*-hexane/EtOAc 85:15). ^1^H-NMR (acetone-d_6_, 400 MHz) δ (ppm): 2.43 (s, 3H), 3.97 (s, 3H), 3.98 (s, 3H), 7.11 (s, 1H), 7.17 (ddd, 1H, *J* = 8.4, 2.2, 1.2 Hz), 7.23 (dd, 1H, *J* = 12.1, 2.1 Hz), 7.28 (td, 2H, *J* = 8.7, 3.5 Hz), 7.50–7.57 (m, 3H), 7.80 (dd, 1H, *J* = 8.1, 2.1 Hz), 9.01 (d, 1H, *J* = 2.0 Hz). 13C-NMR (acetone-d_6_, 100 MHz) δ (ppm): 15.75, 56.65, 56.69, 115.11 (d, *J* = 18.9 Hz), 115.20, 118.18 (d, *J* = 18.1 Hz), 123.76 (d, *J* = 3.0 Hz), 125.60, 127.18 (d, *J* = 3.4 Hz), 128.48. 129.47, 130.71, 131.82, 132.26, 133.13 (d, *J* = 6.6 Hz), 133.83 (d, *J* = 6.3 Hz), 134.68, 139.71, 142.83, 148.56 (d, *J* = 3.6 Hz), 148.67 (d, *J* = 3.2 Hz), 152.84 (d, *J* = 245.8 Hz), 153.48 (d, *J* = 244.7 Hz), 168.15, 168.32. HPLC analysis: retention time = 11.015 min; peak area, 99%. Elemental analysis for C_25_H_19_F_2_NO_4_ calculated: % C, 68.96; % H, 4.40; % N, 3.22; found: % C, 68.97; % H, 4.41; % N, 3.21.

#### (Z)-4-{[4,4′′-Dichloro-(1,1′:4′,1′′-terphenyl)-2′-yl]methylene}-2-methyloxazol-5(4H)-one (20h)

Yellow solid, yield: 7% (18.1 mg) from **19h**. *R_f_* = 0.18 (*n*-hexane/EtOAc 95:5). ^1^H-NMR (acetone-d_6_, 400 MHz) δ (ppm): 2.43 (s, 3H), 7.05 (s, 1H), 7.46 (AA′XX′, 2H, *J*_AX_ = 8.7 Hz, *J*_AA′/XX′_ = 2.3 Hz), 7.54–7.59 (m, 5H), 7.79 (AA′XX′, 2H, *J*_AX_ = 8.8 Hz, *J*_AA′/XX′_ = 2.4 Hz), 7.85 (dd, 1H, *J* = 8.1, 2.0 Hz), 9.06 (d, 1H, *J* = 2.0 Hz). 13C-NMR (acetone-d_6_, 100 MHz) δ (ppm): 15.73, 127.95, 129.45 (2 C), 129.54 (2 C), 129.80, 130.03 (2 C), 131.10, 131.83, 132.34, 132.43 (2 C), 134.42, 134.65, 134.95. 139.14. 139.61, 140.20, 143.32, 168.01, 168.57. HPLC analysis: retention time = 12.830 min; peak area, 95%. Elemental analysis for C_23_H_15_Cl_2_NO_2_ calculated: % C, 67.66; % H, 3.70; % N, 3.43; found: % C, 67.90; % H, 3.71; % N, 3.44.

### Biological evaluation

#### MAGL-inhibition assay

Human recombinant MAGL, and 4-nitrophenylacetate substrate (4-NPA) were from Cayman Chemical. The IC_50_ values for compounds were generated in 96-well microtiter plates. The MAGL reaction was conducted at RT at a final volume of 200 µL in 10 mM Tris buffer, pH 7.2, containing 1 mM EDTA. A total of 150 µL of 4-NPA 133.3 µM (final concentration = 100 µM) was added to 10 µL of DMSO containing the appropriate amount of compound. The reaction was initiated by the addition of 40 µL of MAGL (11 ng/well) in such a way that the assay was linear over 30 min. The final concentration of the analyzed compounds ranged for **CAY10499** and **JZL-184** from 10 to 0.00001 µM and for the synthesised compounds from 200 to 0.0128 µM. After the reaction had proceeded for 30 min, absorbance values were then measured by using a VictorX3 PerkinElmer instrument at 405 nm. Two reactions were also run: one reaction containing no compounds and the second one containing neither inhibitor nor enzyme. IC_50_ values were derived from experimental data using the Sigmoidal dose–response fitting of GraphPad Prism software as reported earlier^16^. To remove possible false positive results, for each compound concentration a blank analysis was carried out, and the final absorbance results were obtained detracting the absorbance produced by the presence of all the components except MAGL in the same conditions.

#### MAGL preincubation assay

The MAGL reaction was conducted at RT at a final volume of 200 µL in 10 mM Tris buffer, pH 7.2, containing 1 mM EDTA. A total of 150 µL of MAGL (11 ng/well) was added to 10 µL of DMSO containing the appropriate amount of compound. After 0, 30, and 60 min of incubation time the reaction was initiated by the addition of 40 µL of 4-NPA 500 µM. The enzyme activity was then measured according to the procedure described above.

#### MAGL dilution assay

The enzyme (880 ng in 75 µL of Tris buffer, pH 7.2) was incubated during 60 min at RT with 5 µL of compound **20b** (concentration of 10 µM in the mixture) dissolved in DMSO. The MAGL-inhibitor mixture was then diluted 40-fold with the buffer. After 15 min of incubation, the reaction was initiated on a 160 µL aliquot by the addition of 40 µL of 4-NPA 500 µM and the enzyme activity was measured according to the procedure described above.

#### FAAH inhibition assay

The IC_50_ values for compounds were generated in 96-well microtiter plates. The FAAH reaction was conducted at RT at a final volume of 200 µL in 125 mM Tris buffer, pH 9.0, containing 1 mM EDTA. A total of 150 µL of AMC arachidonoyl amide 13.3 µM (final concentration = 10 µM) was added to 10 µL of DMSO containing the appropriate amount of compound. The reaction was initiated by the addition of 40 µL of FAAH (0.9 µg/well) in such a way that the assay was linear over 30 min. After the reaction had proceeded for 30 min, fluorescence values were then measured by using a VictorX3 PerkinElmer instrument at an excitation wavelength of 340 nm and an emission of 460 nm. Two reactions were also run: one reaction containing no compounds and the second one containing neither inhibitor nor enzyme. IC_50_ values were derived from experimental data using the Sigmoidal dose − response fitting of GraphPad Prism software as reported earlier^19^. To remove possible false-positive results, for each compound concentration, a blank analysis was carried out, and the final fluorescence results were obtained detracting the fluorescence produced by the presence of all the components except FAAH in the same conditions.

#### Cell viability assay

COV318, OVCAR-3 (from ATCC) and *h*MSC (from AB Cell-Bio) were maintained at 37 °C in a humidified atmosphere containing 5% CO_2_ accordingly to the supplier. Normal (1.5 × 10^4^) and tumor (5 × 10^2^) cells were plated in 96-well culture plates. The day after seeding, vehicle or compounds were added at different concentrations to the medium. Compound was added to the cell culture at a concentration ranging from 200 to 0.02 µM. Cell viability was measured after 96 h according to the supplier (Promega, G7571) with a Tecan F200 instrument. IC_50_ values were calculated from logistical dose–response curves. Averages were obtained from three independent experiments, and error bars are standard deviations (*n* = 3).

### Molecular modeling

#### Consensus docking studies

The ligand was built by means of Maestro^20^ and was then minimised in a water environment (using the Generalised Born/surface area model) by means of Macromodel^21^. It was minimised using the conjugate gradient (CG), the MMFFs force field, and a distance-dependent dielectric constant of 1.0 until they reached a convergence value of 0.05 kcal Å^−1^ mol^−1^. Nine different docking procedures were applied and for each docking calculation only the best scored pose was taken into account^22^. The ligand was docked in the human MAGL (3JWE^23^ PDB code) and the humanised-rat FAAH (3LJ7^24^ PDB code). The ligand was docked into the two proteins by using the different docking procedures, then the root mean square deviation (RMSD) of each of these docking poses against the remaining docking results was evaluated by using the rms_analysis software of the GOLD suite. The most populated cluster was then considered and subjected to molecular dynamic (MD) simulations.

#### AUTODOCK 4.2.3

AUTODOCK Tools utilities^25^ were used in order to identify the torsion angles in the ligand, to add the solvent model and assign the Gasteiger atomic charges to proteins and ligand. The regions of interest used by AUTODOCK^26^ were defined by considering the reference ligand as the central group of a grid box of 10 Å in the *x*, *y*, and *z* directions. A grid spacing of 0.375 Å and a distance-dependent function of the dielectric constant were used for the energetic map calculations. By using the Lamarckian genetic algorithm, the docked compounds were subjected to 20 runs of the AUTODOCK search using 2,500,000 steps of energy evaluation and the default values of the other parameters.

#### DOCK 6.7

The molecular surface of the binding site was calculated by means of the MS program^27^, generating the Connolly surface with a probe with a radius of 1.4 Å. The points of the surface and the vectors normal to it were used by the Sphgen program in order to build a set of spheres, with radii varying from 1.4 to 4.0 Å that describe, from a stereoelectronic point of view, the negative image of the site. Spheres within a radius of 10 Å from the reference ligand were used to represent the site. For each docking calculation, DOCK 6.7 calculated 1000 orientations; of these, the best grid scored was taken into consideration. The ligand charge was calculated using the AM1-BCC method, as implemented in the MOLCHARGE program^28^.

#### FRED 3.0

FRED^29^ requires a set of input conformers for each ligand. The conformers were generated by OMEGA2^30–32^. The following modifications to the default settings of OMEGA2 were applied: the energy window was set at 50.0, the maximum number of output conformers was set at 10,000, the time limit was set at 1200, and the RMSD value below which two conformations were considered to be similar was set at 0.3 Å^33^. The region of interest for the docking studies was defined in such a manner that it contained all residues which stayed within 10 Å from the ligand in the X-ray structures. FRED default parameters were used setting the high dock_resolution.

#### GLIDE 5.0

The binding site was defined by a rectangular box of 10 Å in the *x*, *y*, and *z* directions centered on the ligand. The option allowing only the docking of ligands containing a defined range of atoms was deactivated, whereas the GLIDE^34^ defaults were used for all other parameters. Docking calculations were carried out using the standard precision (SP) method.

#### GOLD 5.1

The region of interest for the docking studies was defined in such a manner that it contained all residues which stayed within 10 Å from the ligand in the X-ray structures; the “allow early termination” command was deactivated, while the possibility for the ligand to flip ring corners was activated. For all other parameters, GOLD^35^ defaults were used and the ligands were subjected to 30 genetic algorithm runs. Three docking analyzes were carried out by using three fitness functions implemented in GOLD, i.e. GoldScore (GS), ChemScore (CS) and Astex Statistical Potential (ASP).

#### AUTODOCK VINA 1.1

The input files for the ligand and proteins originated from the AUTODOCK Tools utilities for the AUTODOCK calculations were also used for the AUTODOCK VINA^36^ calculations, including the grid box dimensions. The exhaustiveness parameter was set to 10 and the Energy_range to 1, whereas for all other parameters, AUTODOCK VINA defaults were used.

#### PLANTS

This docking software uses Ant Colony Optimisation, a state-of-the-art global optimisation algorithm to find minima of a scoring function representing favorable complex structures^37^. ChemPLP scoring function was employed to score protein–ligand interactions as well as intra-ligands clash terms. Standard settings for all parameters were used for the scoring function as well as the optimisation algorithm (search speed setting: “speed1”). The regions of interest used by PLANTS^37^ were defined by considering the reference ligand as the central group of a grid box of 10 Å in the *x*, *y*, and *z* directions.

#### MD simulations

All simulations were performed using AMBER, version 14^38^. MD simulations were carried out using the ff14SB force field at 300 K. The complex was placed in a rectangular parallelepiped water box. An explicit solvent model for water, TIP3P, was used, and the complexes were solvated with a 20 Å water cap. Chlorine or Sodium ions and were added as counter ions to neutralise the system. Prior to MD simulations, two steps of minimisation were carried out using the same procedure described above. Particle mesh Ewald (PME) electrostatics and periodic boundary conditions were used in the simulation^39^. The MD trajectory was run using the minimised structure as the starting conformation. The time step of the simulations was 2.0 fs with a cutoff of 10 Å for the nonbonded interaction, and SHAKE was employed to keep all bonds involving hydrogen atoms rigid. Constant-volume periodic boundary MD was carried out for 1.0 ns, during which the temperature was raised from 0 to 300 K. Then, 50 ns of constant-pressure periodic boundary MD was carried out at 300 K using the Langevin thermostat to maintain constant the temperature of our system. All the α-carbons of the protein were blocked with a harmonic force constant of 10 kcal/mol Å^2^. General Amber force field (GAFF) parameters were assigned to the ligand, while partial charges were calculated using the AM1-BCC method as implemented in the Antechamber suite of AMBER 14.

#### Binding energy evaluation

The evaluation of the binding energy associated to the two ligand–protein complexes analyzed through MD simulations was carried out using AMBER 14. The trajectories relative to the last 50 ns of each simulation were extracted and used for the calculation, for a total of 50 snapshots (at time intervals of 1 ns). Van der Waals, electrostatic and internal interactions were calculated with the SANDER module of AMBER 14, whereas polar energies were calculated using both the Generalised Born and the Poisson − Boltzman methods with the MM-PBSA module of AMBER 14. Dielectric constants of 1 and 80 were used to represent the gas and water phases, respectively, while the MOLSURF program was employed to estimate the nonpolar energies. The entropic term was considered as approximately constant in the comparison of the ligand − protein energetic interactions.

## Results and discussion

### Chemistry

**Scheme 1.**
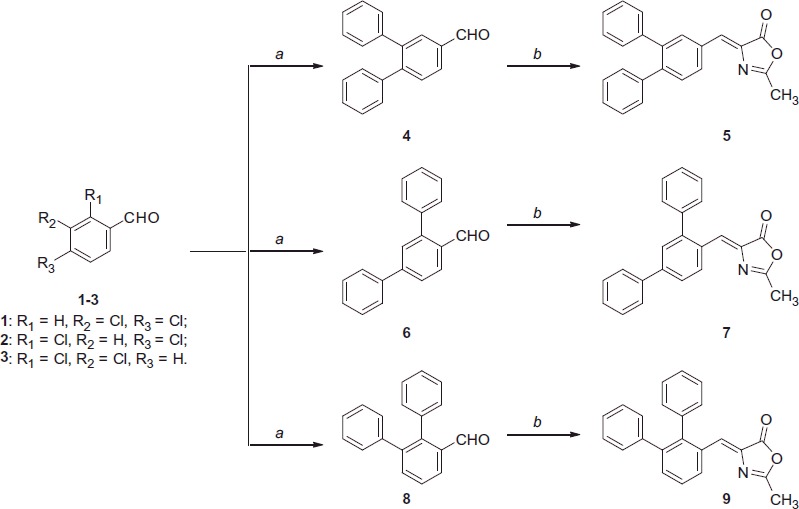
Reagents and conditions: (a) for compound **4**: phenylboronic acid, Pd(OAc)_2_, K_3_PO_4_, TBAB, H_2_O, 125 °C; for compound **6**: phenylboronic acid, Pd(OAc)_2_, PPh_3_, aq. 2 M Na_2_CO_3_, toluene, EtOH, 100 °C; for compound **8**: phenylboronic acid, Pd(OAc)_2_, PPh_3_, aq. 2 M Na_2_CO_3_, toluene, EtOH, 100 °C, then phenylboronic acid, Pd_2_(dba)_3_, Cs_2_CO_3_, Cy_3_P 20% toluene, dioxane, 100 °C; (b) *N*-acetylglycine, Ac_2_O, CH_3_COONa, reflux.

**Scheme 2.**
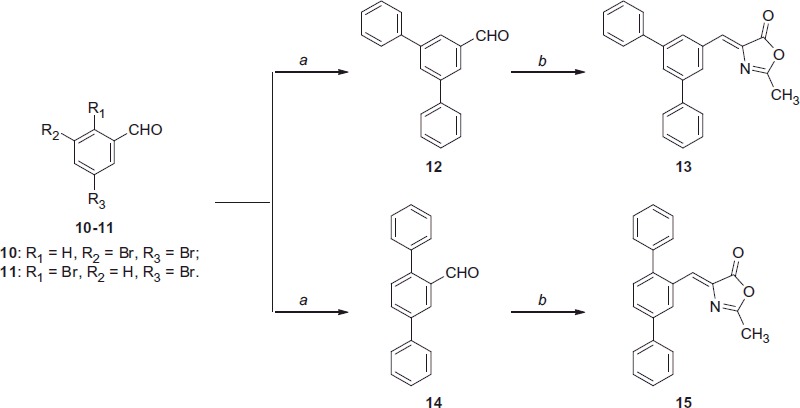
Reagents and conditions: (a) phenylboronic acid, Pd(OAc)_2_, PPh_3_, aq. 2 M Na_2_CO_3_, toluene, EtOH, 100 °C; (b) *N*-acetylglycine, Ac_2_O, CH_3_COONa, reflux.

**Scheme 3.**
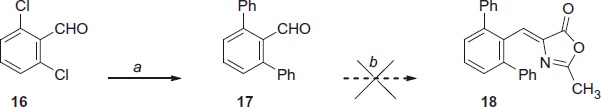
Reagents and conditions: (a) phenylboronic acid, Pd_2_(dba)_3_, Cs_2_CO_3_, Cy_3_P 20% toluene, dioxane, 100 °C; b) *N*-acetylglycine, Ac_2_O, CH_3_COONa, reflux.

The terphenyl compounds were synthesised following the same synthetic strategy applied for the previous series of methyleneoxazol-5(4*H*)-one derivatives, differing only for the first step, in which a double cross-coupling reaction was necessary to replace both the halogen atoms by two phenyl rings in each appropriate dihalo-substituted precursor (Schemes 1 and 2). All the possible combinations of substitutions with phenyl rings in the central aromatic scaffold were explored, with the exception of the 2,6-diphenyl derivative (Scheme 3, see the following chapter for discussion). According to the availability in our laboratory of the starting aldehydes, the coupling reactions were performed on dichloro- or dibromo-substituted compounds and different conditions were chosen on the basis of a preliminary prevision of the reaction outcome, which was highly dependent on the precursor structure. 3,4-Dichlorobenzaldehyde **1** was subjected to a Pd-catalyzed cross-coupling reaction under phosphine-free conditions in water, by using TBAB as the phase-transfer agent, in the presence of potassium phosphate, upon prolonged thermal heating^40^, to give the diaryl derivative **4**. An Erlenmeyer–Plöchl condensation of the substituted aromatic aldehyde **4** with *N*-acetylglycine and sodium acetate in refluxing acetic anhydride gave the final product **5** as the (*Z*)-isomer (Scheme 1). In the case of 2,4-dichlorobenzaldehyde **2**, the classical thermal Pd(PPh_3_)_4_-catalyzed Suzuki conditions gave the desired product **6**. This may appear as a surprising outcome, because the starting material is an aryl chloride which generally is less reactive under these “mild” cross-coupling conditions that are commonly suitable for aryl bromides^41^. Nevertheless, the presence of the aldehyde group in *para* or *ortho*-position to the chloro atoms makes this compound more reactive toward the cross-coupling reaction (Scheme 1). Differently, in the case of 2,3-dichlrobenzaldehyde **3** we failed to perform the double cross-coupling by using simple Suzuki conditions, since ^1^H-NMR analysis revealed the formation of only a mono-substitution product, probably due to the steric hindrance caused by the two chlorine atoms in adjacent positions to the aldehyde group. Therefore, the crude product of the reaction was subjected again to a second cycle of cross-coupling, adopting the Fu-type conditions^42^, which consists in using the more reactive catalytic system comprised of Pd_2_(dba)_3_, together with tricyclohexylphosphine as the catalyst ligand and cesium carbonate as the base (Scheme 1). Both compounds **6** and **8** were then transformed into the corresponding methyloxazol-5(4*H*)-one derivatives **7** and **9** as seen before for the preparation of **5** (Scheme 1).

The synthesis of terphenyl-methyloxazol-5(4*H*)-one compounds **13** and **15** started from bromo-aryl precursors **10** and **11** and cross-coupling, which were performed by adopting Suzuki conditions, allowed the formation of the diphenyl-substituted intermediates **12** and **14** with good yields, respectively (Scheme 2).

Finally, we tried to obtain the last derivative of this series of compounds, which derives from the combination of the two phenyl rings in both of the *ortho-*positions to the oxazolone ring (compound **18**, Scheme 3), starting from the 2,6-dichlorobenzaldehyde **16**. Unfortunately intermediate **17**, which was obtained in high yield from **16** by a Fu-type coupling, did not react under the classical Erlenmeyer–Plöchl conditions, neither by increasing the equivalents of the reagents (*N*-acetylglycine and acetic anhydride: tw0 equivalents) or by extending the reaction time (24 or 48 h). This problem could be ascribed to the steric hindrance of the structure bearing two phenyl rings close to the aldehyde moiety, which hampered the formation of the additional five-membered cycle.

**Scheme 4.**
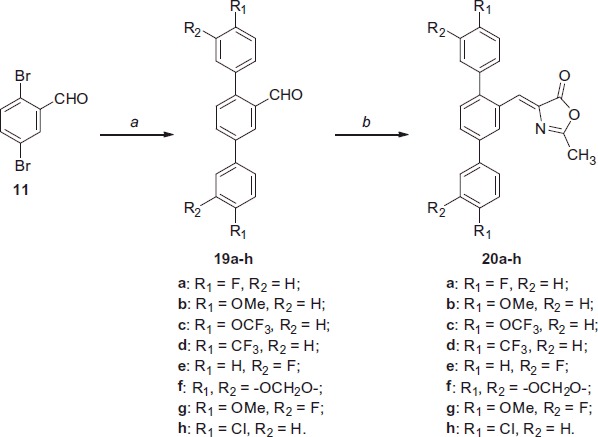
Reagents and conditions: (a) variously substituted phenylboronic acid, Pd(OAc)_2_, PPh_3_, aq. 2 M Na_2_CO_3_, toluene, EtOH, 100 °C; (b) *N*-acetylglycine, Ac_2_O, CH_3_COONa, reflux.

Considering the promising biological activity of compound **15** (see “Biological evaluation” section), which proved to be the most potent *h*MAGL inhibitor among all the possible combinations of terphenyl derivatives synthesised, a series of similar compounds variously substituted on the two peripheral aromatic rings were prepared, in order to investigate the effects of the additional substituents on the enzyme inhibition potency. All these compounds were obtained following the same synthetic pathway adopted for compound **15** (Scheme 2). 2,5-Dibromobenzaldehyde **11** was subjected to a double cross-coupling reaction using the Suzuki conditions with the appropriate boronic acid. Then, intermediates **19a–h** were reacted with *N*-acetylglycine and sodium acetate in refluxing acetic anhydride to give the final compounds **20a–h** (Scheme 4).

### Biological evaluation

The inhibitory effects of the newly synthesised compounds on human isoforms of MAGL and FAAH are reported in Table 1, together with those of reference inhibitors **CAY10499** and **JZL-184**.

**Table 1.** Experimental inhibition activity (IC_50_) on human MAGL and FAAH of the analyzed compounds.


#	Ar_1_	Ar_2_	Ar_3_	Ar_4_	MAGL IC_50_ (nM)	FAAH IC_50_ (nM)	MAGL/FAAH selectivity
**5**	H	Ph	Ph	H	837 ± 18	2321 ± 51	3
**7**	Ph	H	Ph	H	546 ± 20	18161 ± 904	33
**9**	Ph	Ph	H	H	2558 ± 172	20055 ± 1438	8
**13**	H	Ph	H	Ph	457 ± 10	14763 ± 1176	32
**15**	Ph	H	H	Ph	320 ± 10	10860 ± 161	34

**#**	R_1_		R_2_		MAGL IC_50_ (nM)	FAAH IC_50_ (nM)	MAGL/FAAH selectivity
**20a**	F		H		683 ± 50	27989 ± 1306	41
**20b**	OCH_3_		H		348 ± 38	36118 ± 1123	104
**20c**	OCF_3_		H		4194 ± 299	17728 ± 1198	4
**20d**	CF_3_		H		6763 ± 1125	28992 ± 1157	4
**20e**	H		F		628 ± 32	11713 ± 895	19
**20f**	–OCH_2_O–				673 ± 23	23712 ± 1348	35
**20g**	OCH_3_		F		335 ± 2	22311 ± 1239	67
**20h**	Cl		H		476 ± 39	11487 ± 998	24
**CAY10499**					144 ± 4	14.7 ± 0.2	0.1
**JZL-184**					49.8 ± 4.2	3301 ± 205	66

Given the wide range of biological processes regulated by hydrolases, new MAGL inhibitors with a very high level of specificity should be required to minimise mechanism-based toxicities. Dual FAAH/MAGL inhibitors promote cataleptic and drug dependence behaviors in mice that are more reminiscent of direct CB1 agonists^43^, underscoring the importance of maintaining high levels of selectivity to avoid simultaneous blockade of both FAAH and MAGL^44^.

The series of diphenyl-substituted derivatives (**5**, **7**, **9**, **13**, and **15**) revealed that the presence of adjacent phenyl rings in the central scaffold is not ideal, since compound **5** (3,4-diphenyl) and, in particular, **9** (2,3-diphenyl), show the weakest potencies of this initial class. The introduction of further space between the two phenyl rings progressively improves the inhibition abilities of these compounds. In fact, when these substituents are placed in respective *meta*-positions (**7** and **13**), we can observe a significant improvement of the IC_50_ values obtained. This effect is further enhanced when the two phenyl rings are placed in *para*-positions to each other, since compound **15** (2,5-diphenyl) displays the highest MAGL-inhibition potency (IC_50_ = 320 nM) and MAGL/FAAH selectivity (34-fold) of this initial miniseries.

Therefore, we decided to further decorate the phenyl substituents of **15**, and extend the series of 2,5-diaryl-substituted methyleneoxazol-5(4*H*)-one derivatives (**20a–h**). The data reported in Table 1 show that relatively large substituents in the *para*-positions, such as OCF_3_ (**20c**) and CF_3_ (**20d**), do not seem to fit nicely in the enzyme active site since the MAGL-inhibitory activities associated to these compounds are very poor (IC_50_ values of 4–6 µM). The introduction of small halogens, such as fluorine (**20a**,**e**) or chlorine (**20 h**) atoms, or of a dioxolane portion (**20f**) is better tolerated, although the IC_50_ values obtained with these compounds are always higher than that of **15**. Instead, the introduction of *para*-methoxy groups in the peripheral aryl rings, although they do not significantly improve the MAGL-inhibition potency of the resulting compounds (**20b**, **g**) when compared to their unsubstituted counterpart **15**, cause remarkable reductions of their FAAH-inhibitory abilities, thus resulting in a substantial increase in their MAGL/FAAH selectivity. This is particularly evident in **20b**: this compound displays an IC_50_ values of 348 nM against MAGL (similar to that of **15**), together with a noticeable 104-fold selectivity for MAGL over FAAH, which is substantially higher than that shown by both its unsubstituted analog **15** and by reference inhibitor **JZL-184**. Comparing these results with those previously obtained for the monophenyl-substituted derivatives, we can highlight an improvement in terms of MAGL activity and MAGL/FAAH selectivity. In fact, the previously reported compounds showed a MAGL activity in the low micromolar range (IC_50_ = 1.0–2.2 µM) and a MAGL/FAAH selectivity from 15 up to 69-fold^18^. Conversely, the most active compounds of this series (**15**, **20b**, **20g**) displayed IC_50_ values ranging from 320 to 348 nM, thus the presence of two phenyl rings placed in *para*-positions to each other markedly increased the inhibitory potency on MAGL. Moreover, the presence of a methoxy group in *para-*position on both phenyl rings allowed an increase in the MAGL/FAAH selectivity up to 104-fold, as for compound **20b**. Therefore, this compound can be considered as the most promising inhibitor of the present series of methyleneoxazol-5(4*H*)-one derivatives.

**Figure 3.**
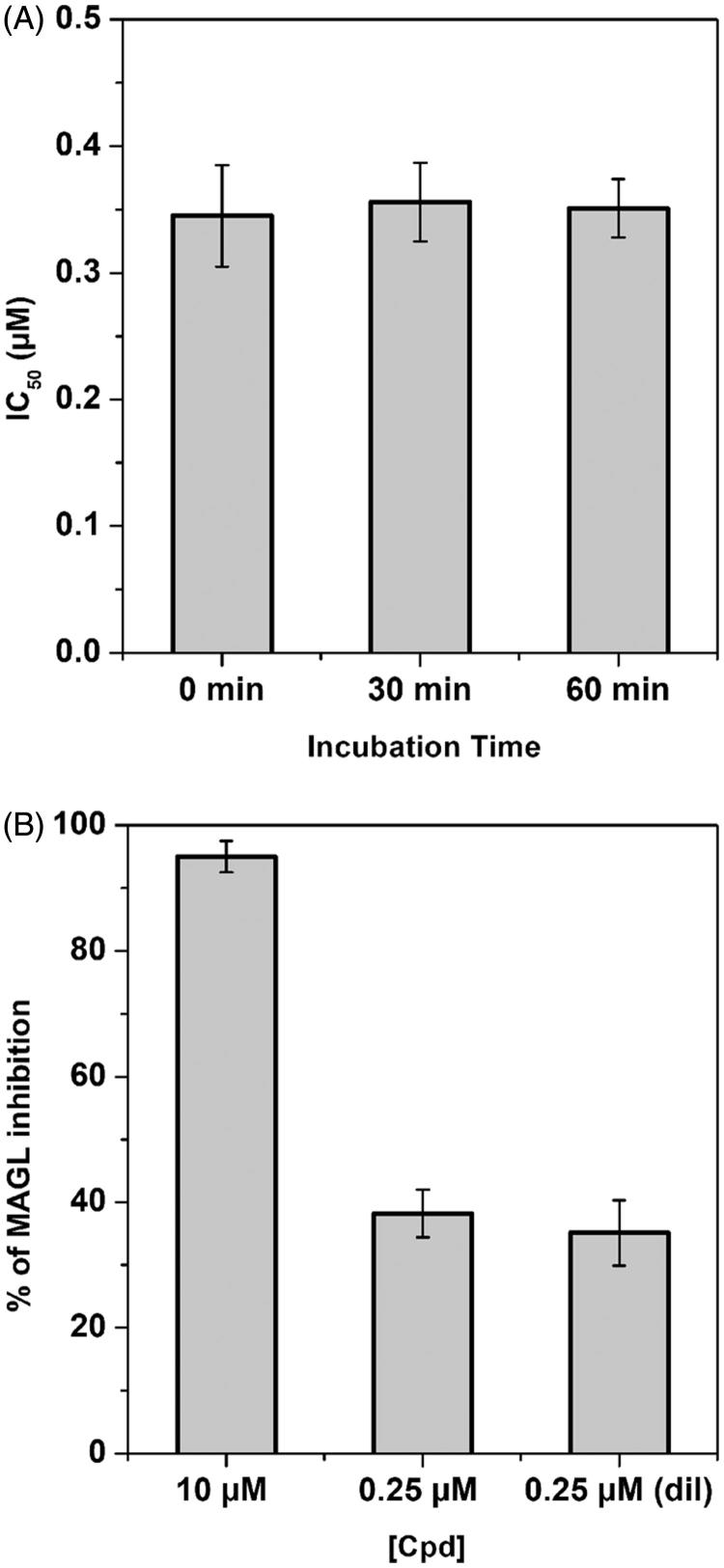
Compound **20b**-MAGL inhibition analysis. (A) IC_50_ (µM) values of **20b** at different preincubation times with *h*MAGL (0, 30 and 60 min). (B) Dilution assay: the first two columns indicate the inhibition percentage of compound **20b** at a concentration of 10 and 0.25 µM. The third column indicates the inhibition percentage of compound **20b** after dilution (final concentration = 0.25 µM).

In order to study the inhibition mechanism of the new reported compounds, the effects of preincubation and dilution in the inhibitory ability of compound **20b** were evaluated. In the preincubation experiments, an irreversible inhibitor will increase its capacity to block the enzyme with increasingly longer incubation times in the presence of enzyme prior to addition of substrate; a constant IC_50_, conversely, supports a reversible mechanism^45^. As expected, compound **20b** did not show any significant increase in its ability to block MAGL activity after 30 and 60 min (Figure 3(A)), supporting that it should be a reversible inhibitor. In the dilution experiments, if **20b** is an irreversible inhibitor, then its inhibition potency should not drop upon dilution, whereas inhibition levels should be substantially reduced upon dilution in presence of a reversible compound. As shown in Figure 3(B), 20b showed reversible inhibition, since the inhibition produced by 10 µM of the compound is significantly higher compared with the inhibition observed upon 40× dilution, which appears similar to that produced by a 0.25 µM concentration of the compound.

Compounds **20b** was also selected for *in vitro* experiments to evaluate its antiproliferative potency on cancer cells. Two human ovarian cancer cell lines, OVCAR3 and CAOV3, were chosen because western blot analysis highlighted an overexpression of MAGL in these two cell lines^17^. The compound produced appreciable inhibition of cell viability for both cell lines, with IC_50_ values of 41.6 µM for OVCAR3 and 23.8 µM for CAOV3. Furthermore, it showed negligible potency against noncancerous human mesenchymal stem cells (*h*MSC, IC_50_>100 µM).

### Molecular modeling studies

**Figure 4.**
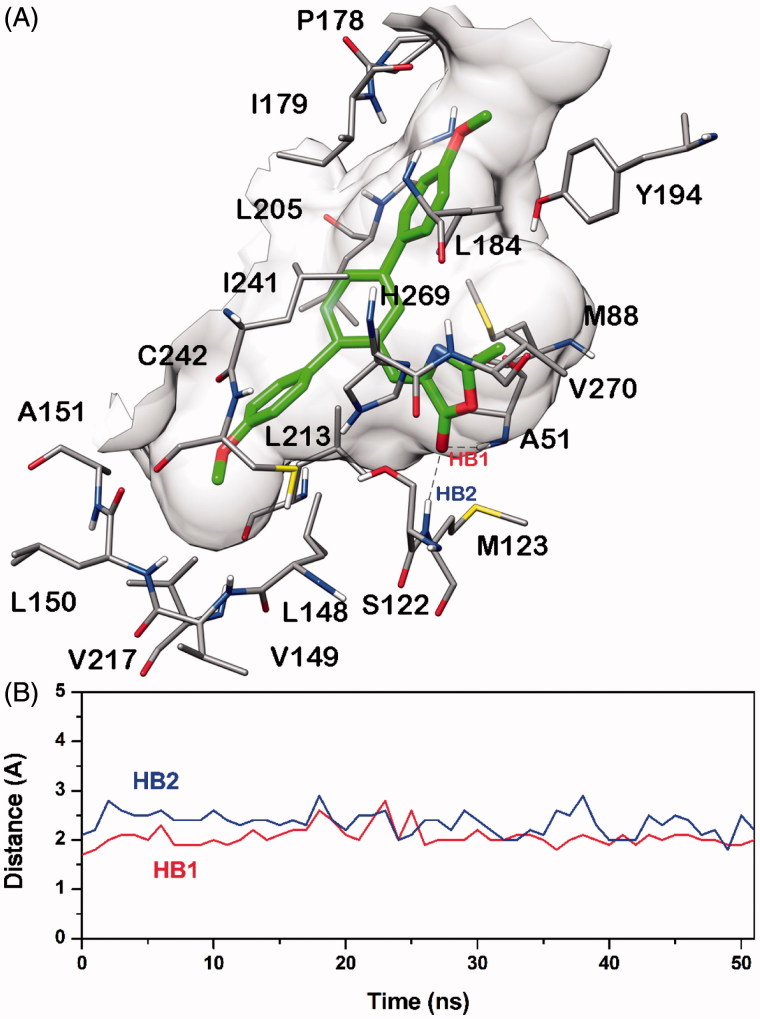
Minimised average structure of compound **20b** docked into MAGL receptor (A) and analysis of **20b**-MAGL H-bond interactions (B). The plot shows the distance analysis for the two H-bonds (i.e. HB1 and HB2).

To suggest a possible binding mode for this class of derivatives, the interaction of compound **20b** with MAGL and FAAH was analyzed by means of docking and MD simulations. This docking analysis helps us to identify the most significant interactions of the compound before the formation of the covalent bond with the catalytic serine, thus highlighting the key points for the ligand recognition. As a first step, a consensus docking method was applied as it is shown to predict the ligand-binding pose better than the single docking programs^46^. By using this kind of approach, one ligand is docked into the target protein by means of different docking procedures. Then, among the different best-ranked poses (originated by the different docking procedures) the pose in common with the largest number of docking procedures is considered as the best docking pose. The **20b**-MAGL and **20b**-FAAH complexes obtained by means of this docking strategy were then subjected to 51 ns of MD simulation with explicit water molecules, as described in the “Material and methods” section. Figure 4(A) shows the main interactions of **20b** with MAGL. The dimethoxyterphenyl fragment occupies the central core of the binding site showing a large number of lipophilic interactions such as those with L148, A151, P178, I179, L184, L205, L213 and L241; whereas the 2-methyloxazolone ring is placed near the catalytic S122 and forms two H-bonds with the nitrogen backbone of A51 (HB1, Figure 4(A)) and M123 (HB2, Figure 4(A)). Interestingly, as shown in Figure 4(B) the H-bonds interactions between the ligand and A51 and M123 displayed a high stability, as they were maintained for the whole MD simulation.

**Figure 5.**
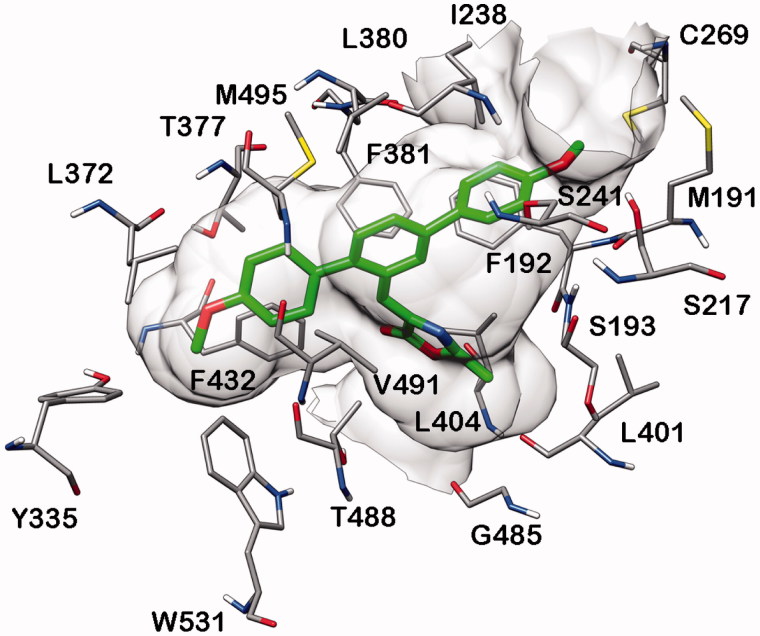
Minimised average structure of compound **20b** docked into FAAH receptor.

As shown in Figure 5, the binding site shape of FAAH does not seem to allow an interaction of the 2-methyloxazolone ring of **20b** in proximity to the catalytic region of the enzyme. The ligand shows a binding disposition that is completely different from that observed in the MAGL binding site, and the 2-methyloxazolone ring is ∼10 Å away from the catalytic S241. Furthermore, the compound does not form any H-bonds with the protein and the ligand is stabilised only by lipophilic interactions with F192, I238, L380, L404 and F432.

In order to further analyze the interaction of **20b** into MAGL and FAAH, the two MD trajectories were further analyzed through the MM–PBSA method^47^, which has shown to accurately estimate the ligand–protein energy interaction^48–50^. This approach averages contributions of gas-phase energies, solvation free energies, and solute entropies calculated for snapshots of the complex molecule as well as the unbound components extracted from MD trajectories, according to the procedure fully described in “Material and methods” section. The MM-PBSA results (Table 2) suggested that the interaction of **20b** with the MAGL binding cavity was more stable by ∼8 kcal/mol than its interaction with FAAH and this energy difference was mainly determined by the lack of strong electrostatic interactions into the FAAH-binding site.

**Table 2.** MM-PBSA results for compound **20b** docked into MAGL and FAAH.

Protein	Ele	VdW	PBsur	PB	ΔPBSA
MAGL	−12.5	−52.2	43.4	−5.2	−26.5
FAAH	−3.2	−50.1	40.2	−5.3	−18.5

ΔPBSA is the sum of the electrostatic (Ele), van derWaals (VdW), polar (PB) and non-polar (PBSur) solvation free energy. Data are expressed as kcal•mol^−1^.

## Conclusions

In summary, we designed and synthesised a new class of terphenyl-2-methyloxazol-5(4*H*)-one derivatives by optimising the benzylidene-2-methyloxazol-5(4*H*)-one scaffold, which was previously identified as a suitable moiety able to efficiently interact with the MAGL-binding site. The reported structural optimisation led to the identification of compound **20b**, which displayed a high MAGL-inhibition activity with an IC_50_ value of 348 nM together with a very good MAGL/FAAH selectivity ratio. Moreover, the biochemical experiments confirmed the reversible properties of this compound and, finally, cell-based assays showed promising cell growth inhibitory activities in the OVCAR-3 and CAOV3 cell lines which overexpress MAGL. Since the *in vivo* possible application of reversible MAGL inhibitors has only recently been explored, mainly due to the deficiency of known compounds possessing efficient reversible inhibitory activities, the present findings constitute an interesting extension to the knowledge of the MAGL inhibition.
